# Practical Implications From European Hospital Pharmacists on Prospective Risk Assessment for Medicine Shortages

**DOI:** 10.3389/fmed.2020.00407

**Published:** 2020-08-05

**Authors:** Nenad Miljković, Eline van Overbeeke, Brian Godman, Milena Kovačević, Alison Anastasi, Tomasz Bochenek, Isabelle Huys, Branislava Miljković

**Affiliations:** ^1^Hospital Pharmacy Department, Institute of Orthopaedic Surgery “Banjica”, University of Belgrade, Belgrade, Serbia; ^2^Clinical Pharmacology and Pharmacotherapy, KU Leuven, Leuven, Belgium; ^3^Division of Clinical Pharmacology, Karolinska University Hospital, Karolinska Institutet, Stockholm, Sweden; ^4^Strathclyde Institute of Pharmacy and Biomedical Sciences, Strathclyde University, Glasgow, United Kingdom; ^5^Department of Public Health and Management, School of Pharmacy, Sefako Makgatho Health Sciences University, Pretoria, South Africa; ^6^Department of Pharmacokinetics and Clinical Pharmacy, University of Belgrade, Belgrade, Serbia; ^7^Central Procurement and Supplies Unit, The Malta Ministry for Health, San Gwann, Malta; ^8^Department of Drug Management, Faculty of Health Sciences, Jagiellonian University Medical College, Krakow, Poland

**Keywords:** medicine shortage, antibiotics, prospective risk assessment, mitigation, substitution, Europe

## Abstract

**Objective:** This study aimed to obtain a comprehensive overview on the perception, attitudes, and experience of European pharmacists with prospective risk assessment procedures in everyday practice, as well as to identify challenges and solutions. This is a follow-up study to the surveys on prospective risk assessment previously carried out within the COST Action 15105 among pharmacists across Europe.

**Methodology:** In-depth interviews were performed using an interview guide comprising 25 questions. Interviews were transcribed *ad verbatim* and imported into NVivo 10 for framework analysis. In NVivo, the interviews were coded through assigning text segments to a responding code from a coding tree, covering the full content of the interviews. Coded text segments were then charted into a matrix, and analyzed by interpreting all text segments per code.

**Results:** In total, 18 interviews were conducted. From the framework analysis, 6 codes and 12 sub-codes emerged. Overall, despite citing specific issues pertaining to its implementation, the interviewees considered multi-stakeholder and multi-disciplinary prospective risk assessment to be essential. While healthcare professionals reported being aware of the importance of risk assessment, they cited insufficient knowledge and skills to be a major obstacle in everyday practice. They also reported inadequate IT support since a paper-based system is still widely in use, thereby complicating data extraction to carry out prospective risk assessment.

**Conclusion:** While prospective risk assessment was found to be valuable, interviewees also found it to be a resource-intensive and time-consuming process. Due to resource constraints, it may not be possible or desirable to conduct prospective risk assessment for every shortage. However, for critical-essential drugs, it is crucial to have a ready-to-use substitute based on risk assessment. Moreover, potential risks of substitutes on patient health should be identified before a shortage occurs and the substitute is dispensed as an alternative.

## Introduction

The impact on patient morbidity and mortality as a result of medicine shortages is well-recognized by all key stakeholder groups including national regulatory authorities globally (1–5). Medicine shortages pose risks ranging from not being able to provide the medicine to being forced to administer a non-optimal substitute, thereby leading to potential medication errors and other complications (6, 7). To combat against shortages, risks must therefore be actively differentiated based on: (i) a shortage's frequency; (ii) the availability and unavailability of treatment options and (iii) providing alternatives but not accounting for a patient's clinical status (8).

Changing treatment when a shortage occurs brings about uncertainties as the order, preparation or dispensing procedures that are needed may also change, which may lead to medication errors (7). This can breed unfamiliarity with the dosing schedules, adverse-effect profiles, and treatment efficacy for therapeutic alternatives among healthcare workers (7, 9, 10). Concerns and unfamiliarity can be enhanced if therapeutic alternatives have not been agreed in advance (11).

A number of surveys have been conducted and individual cases reported to help document the extent of patient harm stemming from changes in treatment as a result of drug shortages. In the USA and Canada, the Institute for Safe Medication Practices (ISMP) provides comprehensive data reports on patient harm caused by shortages (12–14), and in Europe, several healthcare professional associations have conducted surveys showing shortages have a detrimental effect on patient health (4, 5, 15). Similarly, Australian hospital pharmacists have concluded that shortages do prevent patients from being treated with less invasive therapy, such as orally administered forms of the same medicine, which, subsequently, introduce secondary health complications and prolonged costly hospital stays (16).

As a consequence, medication errors caused by shortages need to be thoroughly documented, reviewed, analyzed, and reported as part of safety procedures (7, 17). In addition, possible therapeutic substitutions need to be predetermined and categorized in order to avert potential harm, thereby providing the optimal treatment for a patient in these circumstances (8, 11, 18). A comprehensive risk assessment is the first step in planning for shortages as this allows for a review of all decision making processes in a healthcare setting where risks of patient harm stem from numerous aspects of treatment (19, 20).

Risk assessment is also seen in medicine-shortage reporting systems, not only to assess the critical need for a medicine when there are shortages but also for the institution's ability to continue to provide patient care. This is illustrated by the system in Australia where the criticality of a medicine in a possible shortage situation correlates to its impact on patient's health and accordingly registered in the Medicine's Watch List (21, 22). Similarly, the European Medicines Agency (EMA) in its guidance on the detection and notification of shortages, uses impact analysis for a medicine affected by a shortage, providing a list of criteria used to assess potential alternatives and the size of the patient population affected (23). Regardless of the fact as to whether a medicine shortage is deemed critical or not, healthcare professionals (HCPs), and regulatory authorities must identify treatment pathways, including potential rationing, based on critical assessments so that treatment can be undertaken as effectively as possible (24).

Currently, apart from processes involving blood transfusion, chemotherapy, aspects of surgery, medical devices as well as medicine distribution and prescription, prospective risk assessment appears uncommon in healthcare (25–30). However, its usage has been attracting HCPs attention due to progress that has been made in developing systematic measures to prevent medication errors in healthcare settings via promoting prospective risk assessment tools (31–34).

With medicine shortages on the rise (4, 35–37), therapeutic substitution has become one of the most important steps in providing continuity of treatment to a patient (7, 11, 38, 39). Its importance is not only constituted in the substitution itself, but that this process also represents high risk; as such, it may cause patient harm if not properly designed, implemented and regularly evaluated (7). The authors have previously assessed systematic frameworks and legislative processes for mitigating against drug shortages (40), the extent of medicine shortages in Europe, especially in hospitals, (4) and the extent of using risk-assessment procedures in hospitals to help mitigate the impact of shortages (34). However, the authors wanted to build on this given increasing concern with drug shortages across Europe that needlessly worsen the risk of morbidity and mortality when no risk-assessment is in place. Consequently, a series of in-depth interviews were conducted with hospital pharmacists across Europe to address this matter, aiming to obtain a comprehensive overview on their perception, attitudes, and experience in conducting prospective risk assessment procedures in everyday practice as to address increasing shortages. The findings of which are usable to help develop future policies.

## Methodology

Before initiation of this study, the authors received ethical approval from the Institutional Review Board (IRB) of the University of Belgrade, Faculty of Pharmacy for the verbal consent procedure (Nb. 1221/2). Verbal agreement from all interviewees was also obtained.

This interviewing process built on a previous survey on prospective risk assessment conducted within the COST Action 15105 among 34 HCPs from 26 European countries (34). Interviews were carried out with pharmacists and pharmacologists working in hospitals, academia and for national authorities from member countries of the European Cooperation in Science and Technology COST Action 15105 countries (41). Interviewees were selected via the COST Action 15105 network through purposive sampling and snowballing techniques (42). The interviewees indicated their consent to be interviewed by responding to the email. Interviews were conducted in English via teleconference, telephone or face-to-face based on the preferences expressed of the interviewee. After informing interviewees on the purpose of the interview, verbal consent was again obtained before continuing. Each interview was audio-recorded, transcribed *ad verbatim* and anonymized.

An interview guide was designed for the in-depth interviews. The questions were based on the results from a preceding survey on prospective risk assessment (34), and covered contextual, diagnostic, evaluative, and strategic topics regarding risk assessment. The interview guide comprised three segments with 25 questions in total. The first segment addressed familiarity with risk assessments and information sources on how to apply risk assessments in everyday practice within hospitals. The second segment focused on the practical implementation of risk assessment procedures, detecting risks, and data transfer. The last segment explored the impact of risk assessment on prioritizing in the medicine's substitution process and overcoming shortages, which was followed by questions concerning the characteristics of the healthcare setting where the respondent was employed. The interview guide was piloted at the Faculty of Pharmacy, University of Belgrade and University of Leuven, and subsequently refined prior to use in order to enhance the robustness of the questionnaire.

Interview transcripts were analyzed through framework analysis (43, 44) using NVivo (45). The interviews were transcribed *ad verbatim* by one researcher (NM). At the start of the analysis, two researchers (NM and EvO) familiarized themselves with the content of interviews by reading through the transcripts. When the researchers had any difficulties with understanding the transcript, they re-listened to the audio-recording. Several transcripts were subsequently open-coded. Themes that were identified across the interviews resulted in the creation of “codes” representing these themes. Two researchers (NM and EvO) independently used these codes to classify text segments from the transcripts. After the initial coding was carried out, the researchers agreed on the final set of codes and grouped these in a coding tree (Supplementary Material). The final coding tree was subsequently imported into NVivo and used to code all transcripts by assigning text segments to a respective code. After finalizing the coding phase, results were charted into a matrix where codes formed the columns, and interviewee numbers the rows (Supplementary Material). The data in this matrix was subsequently interpreted by identifying relations between and within codes.

## Results

### Interviewee Characteristics

Interviews were carried out with 17 pharmacists and 1 pharmacologist (Table 1), representing 18 European countries (Austria, Belgium, Bosnia and Herzegovina, Croatia, Cyprus, Estonia, Finland, Germany, Greece, Hungary, Ireland, Italy, Latvia, Malta, Romania, Serbia, Switzerland, and the UK). Of these pharmacists, 14 worked in hospitals, 3 for national health authorities, and 1 pharmacologist working both in academia and in a healthcare-certification agency. The majority of interviewees experienced at least one shortage in the week preceding the interview [28.6%], where 35.7 and 50% of them were forced to use more expensive and off-label alternatives, respectively (Table 1).

**Table 1:** Interviewees' hospital characteristics.

**Hospital characteristics; Median [IQR]**	n (%) (*N* = 14)*
**Number of hospital beds; 700 [300–1,700]**	
<500	7 (50%)
500–1,000	0
1,000–1,500	3 (21.4%)
1,500–2,000	3 (21.4%)
>2,000	1 (7.1%)
**Shortage frequency in last week; 2.50 [1–5]**	
1	4 (28.6%)
2	3 (21.4%)
3	3 (21.4%)
5	2 (14.3%)
≥10	2 (14.3%)
**Type of shortages (demand or supply related)**	
All supply	4 (28.6%)
Mostly supply	8 (58.1%)
Supply and demand	2 (14.3%)
**Use of more expensive alternatives**	
Never	0
Rarely	2 (14.3%)
Sometimes	5 (35.7%)
Often	3 (21.4%)
Very often	4 (28.6%)
**Off-label use of alternatives**	
Never	1 (7.1%)
Rarely	3 (21.4%)
Sometimes	7 (50%)
Often	1 (7.1%)
Very often	2 (14.3%)

* *Three hospital pharmacists worked for national health authorities, not in hospitals. One pharmacologist worked in academia and healthcare certification agency*.

### Insufficient Data, Time, Skills, and Funding to Perform Risk Assessment

When developing risk assessment strategies, a majority of interviewees do report using primary methodological data sources that consisted of: (i) consulting existing materials on the Internet, which serves as a tool for accessing scientific publications on risk stratification and therapeutic guidelines; (ii) official websites of national medicine-regulatory bodies in Europe and the USA; (iii) healthcare-professional associations; and (iv) quality and safety information in healthcare departments. However, the interviewees repeatedly underscored that although the guiding principles and data used for risk assessment need to be accurate, independent and objective, this is not always the case: “For the risk assessment, you need to have reliable information, otherwise it's guessing. So, in many fields we are guessing, because either there is no clear information, or the responsible authorities are not willing to give them. Or they have other aims.” (Hospital Pharmacist, Austria).

By and large, interviewees regard the procedure of risk assessment as time-consuming and not appropriate for all shortages. Whereas, a sufficient number of available generic alternatives do not elicit much need for risk assessment; it is typically reserved for those situations with the greatest clinical impact on patients (including anti-infectives and cancer medicines) when deciding on an alternative treatment is more complex.

Time constraints also limit risk assessment resulting in a multidisciplinary approach not always being feasible. Albeit alerts regarding possible drug-drug interactions must be done conservatively to highlight risk and not over-alert (46–48), a lack of time also limits the ability to pay attention to such alerts. Over-alerting is viewed as an issue among the interviewees. In order to avoid “alert fatigue,” they report selective risk assessment as being generally applied that prioritizes risks but may miss alerts in the process.

Based on their experience, interviewees also report financial restraints as being a further obstacle to applying risk assessment more frequently: “Increasingly getting attention with risk assessments as an incident reporting. Still underfunded—manual lifting issues get more funding. The risk assessment is considered as part of the overall change management documentation (who needs to know, who needs to be consulted).” (Hospital Pharmacist, Ireland).

Training to be able to access, analyze and use data still seems to be an issue. Interviewees from Croatia, Bosnia and Herzegovina, and Hungary emphasized a lack of HCPs possessing sufficient knowledge on risk assessment to be able to adequately perform risk assessment. An interviewee from Ireland also commented that HCPs need better training on handling data for risk assessment and risk assessment itself.

The timeliness of data availability is also a concern. Even when access to data becomes available, it is often too late, as emphasized by an interviewee from Switzerland. This is borne out by the fact that only when reaching out for medicine data on drug availability does such data become accessible. Doubts have also been conveyed on the ability to conduct risk assessment. As underscored by an interviewee from Serbia in particular, essential information on the duration of shortages, as provided by updates from wholesalers and the industry, is often unavailable.

The importance of risk assessment and its benefits for patients, helping to achieve a better state of preparedness for potential shortages, needs to be explained more fully to HCPs. As reported by interviewees from Estonia and Belgium: “I think awareness of the fact and the knowledge of the risk assessment, that's crucial to me. […] as soon as you have people that are aware of risk assessment and see the benefit of it, so that you can use your scarce resources for the best, it's just bringing people together.” (Hospital Pharmacist, Belgium). Moreover, it was also specifically noted that awareness should be extended beyond the pharmacists themselves to the health authorities in charge: “If we are the only ones who think that this is important, this is not going to lead anywhere. We can only discuss this about it among ourselves.” (Hospital Pharmacist, Austria).

Even though there may be a general understanding of the need for risk assessment in mitigating medicine shortages, “the evidence on the impact of shortage of a drug and its extent is missing, there is no data” (Hospital Pharmacist, Hungary). Data on the effectiveness of applied risk assessment procedures is therefore found to be lacking, thereby complicating the evaluation of the real impact that risk assessment has as a mitigation tool. As a result, even communicating the effective results of implementing risk assessment based on data is a challenge, as “it should be done in writing, so actual impact can be seen and prevented” (Hospital Pharmacist, Croatia).

### Insufficiently Structured Approaches Leading to Wide Variation in Risk Assessment

There is a distinct opinion expressed and shared among interviewees concerning an apparent gap in responsibility for “what to do and when you are empowered to make an assessment and then take the action” (Hospital Pharmacist UK). Generally, as reported, hospital pharmacists perceive all HCPs as needing better training on handling data for risk assessment and the risk assessment itself. In Belgium, Bosnia and Herzegovina, Cyprus and Romania, no information is available in this area. Given this feedback, either the data is missing or the guiding documents on shortage mitigation plans and supporting actions are not clearly stated by the stakeholders, both of which are concerns to be addressed.

Interviewees also view a lack of a systematic approach and the option of using electronic databases in performing risk assessment to be a concern: “risk assessment is made individually, based on experience, SmPC (e.g., indications), availability of drugs, prices and outcomes (side effects). It is not documented and follow up rarely happens.” (Hospital Pharmacist, Hungary). Furthermore, the need for IT support is essential for assessment and data dissemination as noted by one interviewee from Austria: “When you have electronic prescribing, implemented in your hospital, you have much more opportunities to control. If you don't it's more difficult you know. You have to talk to everybody, to have more meetings, then you have to say, please do this and you hope that they do that, but you are not sure, you have no technical instruments to help you in the process.” (Hospital Pharmacist. Austria).

As highlighted by an interviewee from Italy, the reactive nature of a retrospective risk assessment—such as with Root Cause Analysis (RCA)—seems to be not as preventative for shortages when compared to the prospective form as Failure Mode and Effects Analysis (FMEA). It is therefore possible to better comprehend and prioritize risks in time and promptly react. In particular, prospective risk assessment is also viewed as a tool which creates faster pathways for medicine substitution before an actual shortage occurs, thereby bringing about tangible clinical benefits for patients. Although interviewees report risk assessment needing to be done in advance, in order to be pro-actively ready with possible solutions and the risks of providing a substitute to patients: “risk assessment needs to happen before an alternative goes to the wards” (Hospital Pharmacist, UK), it is still widely reported that risk assessment is carried out only when the shortage and the need do so occurs.

Interviewees asserted that the risk assessment needs “to be some kind of independent [sic]” and not tailored in the sense that “everyone conducts the assessment for him/herself while it should be the other way around” (Hospital Pharmacist, Germany). Moreover, they point out that risk assessment should be conducted in a systematic and standardized manner in order to not merely solve current problems related to shortages, but to help minimize future problems and equip HCPs as well with the necessary skills to act more efficiently the next time a shortage does occur.

A noted concern that needs to be specifically addressed is that applied risk assessment seems not always to follow scientific rigor: “My experience is that many people use the risk assessment in the way they want to use it so it's in many cases it's more politics than really, what is should [sic] be like science.” (Hospital Pharmacist, Germany). On this basis, as an interviewee from Finland commented, the risks not only need to be clearly defined, but multidisciplinary teams must take the patient's perspective into account throughout the assessment process.

Depending on the complexity of the substitution, interviewees from Belgium and Germany reported that a structured decision making process occurs concerning medicine substitution within Drug and Therapeutics Committees (DTCs). Whereas, in Italy, procedures appear to be more applied to compounding, as well as automated production and dispensing systems compared to medicine shortages. When a generic substitution occurs, pharmacists provide data quickly on an alternative into electronic prescribing systems. Similar to the situation in Estonia, if a therapeutic alternative is needed, physicians are involved into the discussion within DTCs and nurses are fully informed of the substitute's administration patterns.

In Croatia, Cyprus, Estonia, Hungary, Serbia and Switzerland, risk assessment is conducted in everyday practice on a case-by-case basis. In Belgium, Hungary, and Serbia and Belgium risk assessment takes on differing forms depending on the severity of the medicine shortage. In Serbia, for example, the healthcare system performs assessments quickly but only if an appropriate alternative exists at the immediate healthcare-facility level; if not, the Serbian Ministry of Health and National Health Insurance Fund intervene and propose measures based on their own risk assessment. Depending on the patient population size affected and the duration of the shortage, DTCs in Hungary take differing measures to cope with carrying out a quick risk assessment that provides outputs in terms of whether the needed generic substitution is feasible. Otherwise, the DTCs and national task force must be involved, including a more complex analysis and therapeutic proposals. Risk assessment is incorporated into a healthcare facility's accreditation and certification system in Bosnia and Herzegovina as part of the overall safety management plan, while health, safety and quality officers in Cyprus provide training on risk assessment applications in hospital.

An interviewee from Austria (HCP and clinical risk manager) applies a clinical incident reporting (CIR) system based on general risk assessment procedures (such as FMEA) in the overall quality management via electronic tools, but not in the area of medicine shortages. The belief was also expressed that risk assessment is currently linked more to procurement and should be performed prior to tendering procedures in order to incorporate issues such as the criticality of a medicine, increasing safety stocks and possible case-by-case analysis, as part of decision making going forward: “I think pharmacy is in a very good position to make these decisions, but that should be done in conjunction with the managers of the hospital.” (Hospital Pharmacist, UK).

### Unclear Cross-Sharing of Outcomes Among Stakeholders

Interviewees expressed their concern in relation to not sharing data on risk assessment outcomes or results among key stakeholders, viewing it as another challenge in spreading a culture of performing risk assessment.

Interviewees from Germany and Malta particularly expressed their concern in relation to not sharing data on risk assessment outcomes or results among stakeholders. Whilst currently not in place, an incident reporting system has been noted as being necessary in Italy in order that HCPs from all healthcare facilities may share data among themselves, as to be able to perform risk assessment accordingly. In the context of having a transparent process of risk assessment, it was noted that “if you want to reduce risks, you need to disclose them and present existing risks to the stakeholders, which calls for action” (Hospital Pharmacist, Switzerland).

Communicating the risk assessments performed appeared to be conducted via various routes. Most of the interviewees stated that the internal communication dealing with medicine shortages, including potential risks, is usually carried out via the intranet, emails, electronic prescribing systems/Computerized Physician Order Entry (CPOE), and sometimes via phone calls. These included email, calls, websites, and other official means of information exchange, which is currently not perceived as efficient by a majority of the interviewees. Nevertheless, it is seen as an important tool in managing shortages successfully due to the time-sensitive nature of the information being communicated. Being of this opinion, however, does not mean that all information is shared equally across cultural lines: “I think we share a lot, but not everyone shares the same information, but we are sharing. I'm not sure if this a culture thing.” (Hospital Pharmacist, Malta) and “It's not really exchange. Maybe, it's still somehow old culture, not to tell, not to say…” (Hospital Pharmacist, Latvia). It was also stated in Italy that the regular exchange of information among stakeholders on incidents provoked by shortages is mandatory and is integrated into their quality assurance systems and enforced through policy measures.

There are also ongoing initiatives by HCP bodies to organize separate channels of communication to better suit their needs building on current concerns and challenges: “I think you have to share the information, that's not discussion about that. However, not everybody needs every information. You need to choose a different language.” (Hospital pharmacist, Austria). Initiatives include organizing lectures for patients in order to provide them with more information on their medical treatment. Overall, the way all stakeholders share information and communicate with each other on detected risks due to shortages should be carried out in a more transparent and structured way.

### Principal Reasons Cited to Implement Risk Assessment and the Factors Determining Its Success

Interviewees cite a number of reasons to implement risk assessment; chiefly among these include (i) providing as many patients as possible with adequate medical treatment; (ii) assuring the treatment's continuity; and (iii) avoiding medical errors and increasing the patient's safety through incorporating risk assessment into quality measurement systems.

**Figure 1:**
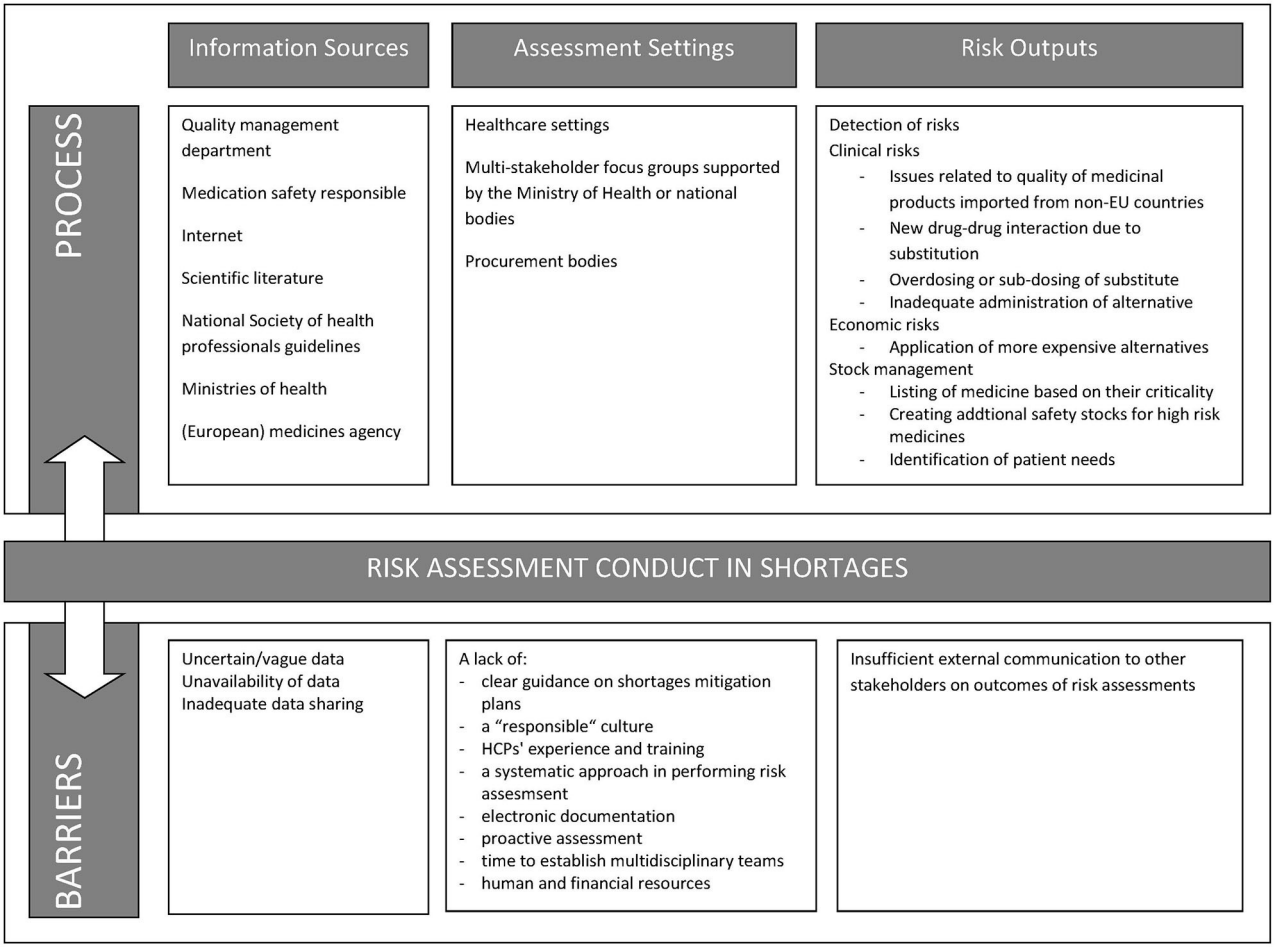
Barriers along the process of risk assessments.

The principal factors determining the success rate of risk assessments in a medicine shortage were knowledge of risk assessment techniques, data reliability, and communicating risk assessment output among stakeholders. The barriers also faced when applying risk assessment were identified throughout the interviews in Figure 1. Table 2 also lists solutions and challenges to address these factors and barriers.

**Table 2:** Challenges and solutions for overcoming barriers in applying risk assessment in shortages.

**Factors**	**Challenges**	**Solutions**
**Resources for risk assessment**		
Financial resources	Difficulties in obtaining funding for hiring experts in risk assessment, train healthcare professionals in this field and provide additional working hours specifically dedicated to shortages	Provide sufficient funds to carry out risk assessment in order to prevent more costly drug adverse effects that need to be mitigated due to improperly mitigated shortages
Human resources	Lack of experts in the field of risk assessment among healthcare professionals	Provide training on risk assessment techniques with real case examples in shortages
Data accessibility and sharing	Not having timely, precise and reliable information and feedback from all stakeholders involved in shortages processes	Provide means for open and transparent communication across healthcare sectors including local healthcare setting environment
**Risk assessment methodology**		
Retrospective risk assessment	Reactively treating emerging risks	Using retroactive approach only for recording trends and not for overall risk analysis
Prospective risk assessment	Not being able to incorporate all potential risks that might occur within a holistic risk assessment process	Involve a thorough sub-processes analysis in order to capture an overall risk assessment
Multidisciplinary/multi-stakeholder perspective in risk assessment	Lack of willingness for healthcare professionals to take the role in multidisciplinary work on shortages including insufficient stakeholder engagement	Increase safety culture and provide educational incentives
**Risk assessment implementation**		
Internal risk assessment output communication	Lack of IT infrastructure and internal standard operating procedures in place	Introduce clear SOPs and improve IT infrastructure
External risk assessment output communication	Separate risk assessments conducted by stakeholder	Coordinated multi-stakeholder task group activities with shared risk outputs
Risk assessment output communication to patients and/or caregivers	Insufficient or no transfer of information on a substitute's efficiency or adverse events profile to patients	Introduce clear SOPs and educate healthcare professionals on communication strategies with patients and/or caregivers during shortages

### Associated Major Risks Stemming From Medicine Shortages

Most interviewees (particularly from Latvia, Belgium, and Croatia) recognized risks related to overdosing, or sub-dosing of patients, after substitution taking place due to differences in the alternative's strength and the pharmaco-technological form applied. Furthermore, interviewees agreed that new drug-drug interactions may emerge after introducing a substitute. As stated by an interviewee from Serbia, inappropriate medicine substitution that is solely led by availability may result in worsening health outcomes in general or increased antimicrobial resistance, which is a global concern (49–52). It was also noted in the interviews that patients might not be properly informed about the alternatives and could be therefore less compliant to treatment. The risks emerging from a substitution also include the non-familiarity of HCPs with administration patterns, which may also bring about adverse events and suboptimal treatment.

## Discussion

Beyond placing undue financial burdens on a healthcare system and its patients, medicine shortages also bear a significant impact on patient treatment, affecting their health outcomes via delaying or interrupting treatment or enforcing application of less efficient treatment (53). Hospital pharmacists, representing interviewees in this study, provided their insight into shortages, proposing potential solutions to alleviate their widespread, and overarching effects.

### Engagement Needed to Foster Risk Assessment

Overall, the findings point to the need for wider multi-stakeholder engagement and guidance concerning ways to mitigate shortages in the future. This includes risk assessment in line with guidance from key groups in the US to manage the impact and forces that drive shortages (7, 17), particularly because information on shortages published on respective national websites is typically outdated. In addition, interviewees generally learnt about shortages from wholesalers and manufacturers after it was too late to mitigate the shortage successfully. This pronounced lack of data sharing between stakeholders within and across countries to aid the initiation of risk assessment is in stark contrast to EMA recommendations for national authorities and stakeholders to mitigate shortages (54).

It would appear that even mandatory (i.e., enforced by the law) information to be shared on shortages are not equally and timely disseminated among stakeholders (55, 56). An early warning system has been recognized widely by HCPs to be crucial in terms of providing a timely reaction to shortages (57). Supply-chain actors may enforce a more proactive mitigation of shortages, which would entail and foster more comprehensive communication with National Competent Authorities (NCAs) and HCPs (53).

Moreover, there is a distinct concern regarding medicine shortages when detailed management and mitigation guidance is lacking from higher authorities (58, 59). The non-harmonized approach is reflected in the manner in which shortages are managed including risk assessment in some European countries for particular groups of medicines, generally life-saving ones. Research into what country takes what approach is largely still novel, at least for Europe. More to the point, as found in recent research, applied risk assessment lacks such prominence in practice that there is a reported general state of being unaware of any governmental or non-governmental strategies of risk assessment to tackle shortages (34). In spite of the European Association of Hospital Pharmacists' (EAHP) Statements of Hospital Pharmacy underscoring the necessity for contingency plans to mitigate shortages, any such strategy in Europe is still uncommon (60). Whereas, there is a conscious need among HCPs regarding how assessment may detect possible medicines shortages and lessen their impact on patient health, there is as of yet inadequate promotion of applying European-wide risk assessments in a prospective manner (34).

Risk assessment, as a concept in increasing preparedness for medicine shortages and ameliorating their health impact, is usually conducted by the manufacturers addressing production and quality control risks (6). The EMA's Pharmacovigilance Risk Assessment Committee (PRAC), on the other hand, stipulates a wider multi-stakeholder commitment assuring protection, and promotion of public health (61). In other words, risk assessment in shortages is also of a public health concern. Since, healthcare professionals, including pharmacists, participate in the work of NCA's, and PRAC under the auspices of EMA, they should be equipped with the skills necessary to conduct risk assessment, including sharing their output and fostering multi-stakeholder engagement (62).

Doing so should also be within the remit of the NCAs that should closely coordinate via addressing issues related to data sharing, conducting best practices, and developing strategies, all in order to provide a safe environment for medicine application across Europe and throughout the European regulatory system for medicines (63). Nonetheless, the EMA's role in coordinating the work of expert groups recruited from scientific experts, healthcare professionals, and patients should also here be recognized. These groups may propose guidelines and scientific advice built on experience and expertise in order to supervise medicine safety (63).

### Structured Risk Assessment Needed

The ASHP guidelines state that pharmacists should carry out available stock assessment in relation to usage patterns and duration of shortages as well as undertake impact assessments embodying all the elements of risk assessment (7). However, a number of the interviewees in this study expressed the view that risk assessment is mostly conducted in medicine through the purchasing process rather than in medicine substitution with therapeutic assessments typically coordinated by hospital DTCs. This is because therapeutic assessments are not generally characterized as risk assessment among hospitals in Europe; however, they do include scenario-based assessments whereby HCPs or task-force groups assess the current situation of shortages, predict future shortages, and the potential to mitigate them to be better prepared in the event of likely shortages in accordance with ASHP guidance (7). Overall, there is a need for clear guidelines describing risk assessment itself building on national guidance (7, 17, 24) as evidenced by the interviewees' agreeing that risk assessment helps with the prioritization of both patients' needs as well as medicines that must be quickly substituted. This should be conducted especially for critical-essential drugs in line with the guidance that data on shortages with a critical patient impact are separately listed with proposed actions for management (64). Moreover, potential risks for patient health, which a substitute brings, should be listed before a shortage actually occurs and a medicine is dispensed as an alternative especially as specific monitoring features are not typically listed on shortages databases (1, 7, 17, 56). In line with this, the interviewees repeatedly reiterated that risk assessment is of crucial importance for critical medicines, or medicines needed to treat patients in critical conditions. Moreover, medicine criticality is based on the availability of alternatives, indication, and the patient population in the patient's respective healthcare settings. This should be taken into account in procurement processes by creating additional safety stocks with those medicines that bear the “highest” risk factors.

### Facing Challenges and Constraints in Carrying out Risk Assessment

A typically distinct lack of time and financial resources is an ongoing issue when seeking to apply risk assessment strategies in hospitals, which is hindered by a lack of publications characterizing and quantifying the impact of shortages on HCP practices, patient health, and overall costs as well as practices leading to them (17). Such an absence of informative materials needs to be urgently addressed, although recognizing that there are only a limited number of experts in the field, it can be challenging to change the mindset of HCPs who have grown accustomed to longstanding forms of practice. However, better patient outcomes are indeed associated with a higher culture of patient safety, as influenced by HCPs' beliefs and values (65, 66). Furthermore, not having a digitized system certainly limits conducting risk assessment as data first needs to be compiled from non-digital sources, a task that is both time-consuming and challenging, prior to being applied into analyses.

### Enhancing Communication Among Stakeholders Throughout Risk Assessment

One of the most important aspects of prospective risk assessment emphasized by the interviewees is communication between the stakeholders and HCPs involved in this process, which is currently subject to variation. DTCs and dedicated shortage task force teams are considered essential in trying to communicate data efficiently in healthcare settings and avoid problems encountered by outdated information (67). There would also seem to be insufficient reference among current EMA guidelines to risk assessment applicable for HCPs, as well as current EMA guidance on the data needed by HCPs to help mitigate against shortages tending to be more reactive than proactive (56), reflected in the interviewees' concern about not possessing the necessary knowledge and skills to perform risk assessment. As a result, the majority of the interviewees underlined the need to apply such proactive approaches in the future. In particular, as stipulated by the US FDA, adjusting e-health patient records, recoding IT systems to respond to available substitutes, as well as relabeling and repackaging medicines to assist with managing future shortages (17). Alongside this, all implemented treatment protocols in healthcare settings need to go through extensive revision in order to respond to treatment changes/adjustments arising from shortages (17). Having said this, HCPs in our study still consider performing risk assessment ineffective if not supported by their respective national regulatory bodies as well as other associated groups.

There is also the evident critical need to share the outputs of risk assessment to enhance uptake of such strategies along with general communication regarding shortages in accordance with numerous mitigation guidelines across countries (7, 17, 22, 68), which entails improving channels of communication and messages sent to the stakeholders. Moreover, clinical advice should be provided and any other additional information needed to prevent risk for patient health arising from shortages, including data on the duration of action and doses needed to achieve therapeutic outcomes with alternatives. Such advice should be regularly updated and be in line with guidance and activities from regulatory authorities in, as is conducted on a national level in Australia and the USA (7, 22, 24, 64, 69). These activities should help address interviewee concerns where they report delays in, or interruption of, treatment forcing them to resort to unsafe, and sub-optimal treatments for patients particularly for cancer patients and emergency medicines, as well as anesthetics and antibiotics (4, 5, 17). Better coordination and information among governmental authorities is key.

There has been growing demand among hospital pharmacists for more timely and accurate information on shortages to better support patient treatment. In order to attain more reliable and accurate information, it would be recommendable that all actors in the supply chain incorporate their own data on shortages into a whole that would better foresee potential shortages on the horizon and communicate them with one another. The immediate advantages of such a measure would be that the individual actors would be able to detect a shortage as it appears (59). Only through a common shared network may information on shortages be used to combat and anticipate them. This may consequently improve patients safety and facilitate shortage mitigation. We have seen this in other situations to improve the quality and efficiency of prescribing of both new and established medicines (70–76).

Ultimately, risk assessment performed on medicine shortages should reach the public in a comprehensive manner and address their concerns. One of the biggest patient concerns is when a medicine will be available in order not to have their care compromised or delayed, especially if a substitute intended to be used may ultimately prove less effective (77, 78). To help address these and other patient concerns, tangible information to patients needs to be provided in a more comprehensive manner.

## Limitations of the Study

Although this study is qualitative in nature and reports chiefly on the current views and perceptions of those who are working as hospital pharmacists in how they mitigate medicine shortages and where they could see improvement in practices, the study does not incorporate other viewpoints from a wider variety of stakeholders involved in addressing medicine shortages on European scale. As such, there is a predefined limitation of what the study may conclude. More comprehensive studies that seek to address risk assessment in order to mitigate medicine shortages must include a wider range of stakeholders; however, such a limitation also points to the path that multi-stakeholder engagement should take in future.

## Conclusion

Prospective risk assessment gained more attention in recent years with patient safety set as a priority. Whilst HCPs are aware of the importance of risk assessment as well as available official sources of information regarding methodology, there is currently a lack of knowledge and skills on how to apply risk assessment in everyday practice. Moreover, the data needed for a proper assessment of the risks posed by medicine shortages is currently not provided in a timely-enough manner, which is essential should successful mitigation be achieved. In future, risk assessment should be carried out more in a prospective fashion so that emerging risks from shortages may be better prevented before they even occur. Notwithstanding, this concept of assessing risk is still not widely practiced in the area of shortages and needs to be urgently addressed to better equip HCPs with the tools they need to manage growing medicine shortages worldwide.

Although the process of prospective risk assessment is often considered to be time-consuming and not appropriate for every single shortage, it is necessary, especially for emergency medicines. In addition, the impact of shortages is not very well-documented. Both these challenges need to be addressed as being able to quickly react to an emerging shortage is considered one of the benefits of prospective risk assessment.

## Data Availability Statement

All datasets generated for this study are included in the article/Supplementary Material.

## Ethics Statement

Ethical approval was obtained from the Institutional Review Board (IRB) of the University of Belgrade, Faculty of Pharmacy including the use of the verbal consent procedure (Nb. 1221/2). Verbal agreement from all interviewees was also obtained.

## Author Contributions

NM designed and carried out the interviews and compiled the first draft and the subsequent iterations of the manuscript. NM and EO performed data analysis and interpretation needed for the manuscript. AA, BG, BM, EO, IH, MK, and TB contributed to critical analysis and interpretation of data as well as revised the manuscript. All authors read, commented on and contributed to the manuscript.

## Conflict of Interest

The authors declare that the research was conducted in the absence of any commercial or financial relationships that could be construed as a potential conflict of interest.
