# Supplementary material for: Practical Implications From European Hospital Pharmacists on Prospective Risk Assessment for Medicine Shortages
Source: Front Med (Lausanne). 2020 Aug 5;7:407. doi: 10.3389/fmed.2020.00407 (PMC7419473; doi:10.3389/fmed.2020.00407)
Supplement: Supplementary file 1 [file Table_1.DOC]

1. **Information on risk assessment**
   1. Sources of information
   2. Information gaps
   3. Information for patients (lay-people)
2. **Experience with risk assessment**
3. **Reasons for applying risk assessment**
   1. Perceptions of risk assessment
   2. Impact of applied risk assessment
4. **Applying risk assessment**
   1. Process of applying risk assessment
      1. Challenges in applying risk assessment
5. **Output of risk assessment**
   1. Detected risks after risk assessment
   2. Ranking risks
   3. Prioritization of patients/patients needs
6. **Communication of information based on risk assessment**
   1. Internal communication
   2. External communication
